# Differential airway resistome and its correlations with clinical characteristics in *Haemophilus-* or *Pseudomonas*-predominant microbial subtypes of bronchiectasis

**DOI:** 10.1186/s12931-023-02562-8

**Published:** 2023-11-02

**Authors:** Xin-zhu Yi, Jun-hao Yang, Yan Huang, Xiao-rong Han, Hui-min Li, Lai-jian Cen, Zhen-hong Lin, Cui-xia Pan, Zhang Wang, Wei-jie Guan

**Affiliations:** 1 Institute of Ecological Sciences, School of Life Sciences, South China Normal University, 55 Zhongshan Boulevard West, Guangzhou, China; 2 State Key Laboratory of Respiratory Disease, National Clinical Research Center for Respiratory Disease, Guangzhou Institute of Respiratory Health, The First Affiliated Hospital of Guangzhou Medical University, 151 Yanjiang Road, Guangzhou, Guangdong China; 3 Department of Geriatrics, National Key Clinical Specialty, Guangzhou First People’s Hospital, South China University of Technology, Guangzhou, China; 4 Department of Pulmonary and Critical Care Medicine, The Second Affiliated Hospital of Guangzhou Medical University, Guangzhou, China; 5 Department of Thoracic Surgery, Guangzhou Institute of Respiratory Disease, First Affiliated Hospital of Guangzhou Medical University, Guangzhou, Guangdong China

**Keywords:** Antibiotics, Resistome, Macrolides, Disease severity, Inflammation, Bronchiectasis

## Abstract

The prevalence and clinical correlates of antibiotic resistance genes (ARGs) in bronchiectasis are not entirely clear. We aimed to profile the ARGs in sputum from adults with bronchiectasis, and explore the association with airway microbiome and disease severity and subtypes. In this longitudinal study, we prospectively collected 118 sputum samples from stable and exacerbation visits of 82 bronchiectasis patients and 19 healthy subjects. We profiled ARGs with shotgun metagenomic sequencing, and linked these to sputum microbiome and clinical characteristics, followed by validation in an international cohort. We compared ARG profiles in bronchiectasis according to disease severity, blood and sputum inflammatory subtypes. Unsupervised clustering revealed a *Pseudomonas* predominant subgroup (n = 16), *Haemophilus* predominant subgroup (n = 48), and balanced microbiome subgroup (N = 54). ARGs of multi-drug resistance were over-dominant in the *Pseudomonas*-predominant subgroup, while ARGs of beta-lactam resistance were most abundant in the *Haemophilus*-predominant subgroup. *Pseudomonas*-predominant subgroup yielded the highest ARG diversity and total abundance, while *Haemophilus*–predominant subgroup and balanced microbiota subgroup were lowest in ARG diversity and total abundance. *PBP-1A*, *ksgA* and *emrB* (multidrug) were most significantly enriched in *Haemophilus*-predominant subtype. ARGs generally correlated positively with Bronchiectasis Severity Index, fluoroquinolone use, and modified Reiff score. 68.6% of the ARG-clinical correlations could be validated in an independent international cohort. In conclusion, ARGs are differentially associated with the dominant microbiome and clinical characteristics in bronchiectasis.

**Supplementary Information:**

The online version contains supplementary material available at 10.1186/s12931-023-02562-8.

## Introduction

Chronic respiratory diseases (CRDs) such as bronchiectasis [1, 2], chronic obstructive pulmonary disease (COPD) [2] and asthma [3] are characterized by perturbed airway microbiome which leads to heightened inflammatory responses [4]. Dysbiosis, characterized by expansion of pathogenic bacteria and reduction in microbial diversity, correlates with the disease severity of bronchiectasis [1] and inflammatory subtypes of COPD [5]. The microbial compositions could also predict the clinical trajectory and outcomes in CRDs such as bronchiectasis [1].

Lower airways are the niche of microbiome, the reservoir of antibiotic resistance genes (ARGs), particularly in patients with structural lung diseases [6–10], prolonged or repeated exposures to antibiotics [11, 12] or acute viral infections [13]. Previous studies have documented the high abundance of ARGs which correlated with bacterial loads in COPD [14] and the shared macrolide-related resistome among bronchiectasis, COPD and asthma [15, 16]. In bronchiectasis, prolonged exposures to azithromycin has been associated with the selection of drug-resistant *Pseudomonas aeruginosa* [17]. Meanwhile, the growing burden of antibiotic resistance because of extensive antibiotic exposures has become concerning globally [18, 19], which could readily result in blunted antimicrobial capacity and confer selective pressures on antibiotic-resistant bacteria [17]. The clinical correlates (disease severity, clinical and inflammatory subtypes) of ARGs in CRDs remain unclear.

Bronchiectasis is characterized by irreversible dilated bronchi which harbor abundant pathogenic bacteria [20]. Patients may experience recurrent exacerbations which frequently require antibiotics and readily acquire antibiotic-resistant bacteria [14, 15]. The clinical and biological characteristics of the human host, including the disease severity and airway inflammatory subtypes could shape the microbiome and ARGs profiles in bronchiectasis. However, no study has directly linked ARGs expression profiles to the clinical heterogeneity of bronchiectasis.

By performing an in-depth metagenomic shotgun sequencing, we profiled ARGs in adults with bronchiectasis via linking sputum microbiota to ARGs, and explored their clinical correlates. We hypothesized that the ARG profiles were influenced by the sputum microbiota taxa and the clinical metrics of bronchiectasis.

## Methods

### Study populations

We prospectively enrolled participants aged 18 years or greater between May 2019 and January 2021 from out-patient clinics of The First Affiliated Hospital of Guangzhou Medical University, a major tertiary hospital specialized in and a national clinical research center for respiratory medicine. All eligible bronchiectasis patients had to be symptomatic (chronic cough, daily sputum production, etc.), remained stable for 4 weeks, and had undergone high-resolution computed tomography within 12 months. We excluded patients with an exacerbation [21] or antibiotic use (except for maintenance low-dose macrolides) within 4 weeks. Exacerbation denoted a significant deterioration of three or more symptoms lasting for ≥ 48 h (cough; sputum volume and/or consistency; sputum purulence; breathlessness and/or exercise tolerance; fatigue and/or malaise; hemoptysis) that required immediate changes in treatment.

Through advertisement, we recruited healthy subjects who had normal chest X-ray and spirometry findings, no lower respiratory tract infections within 4 weeks or uncontrolled systemic diseases.

Ethics approval was obtained from the ethics committee of The First Affiliated Hospital of Guangzhou Medical University (Medical Ethics 2016, the 32th). All participants gave written informed consent.

### Study design

We collected information pertaining to prior antibiotic exposures, co-existing respiratory diseases and concomitant medications from medical charts. We evaluated radiologic severity with modified Reiff score, performed spirometry and sputum cell differentials, and rated the bronchiectasis severity index (BSI) [22]. Co-existing asthma and COPD was diagnosed based on *Global Initiatives for Asthma* [23] and *Global Initiatives for Obstructive Lung Disease* [24], respectively. The BSI was stratified into mild (0–4), moderate (5–8) and severe (9 or higher) categories. Some patients who had experienced exacerbations donated sputum at follow-up visits (exacerbation cohort). Clinical assessments for healthy subjects consisted of clinical history and smoking status inquiry, spirometry, chest X-ray and sputum induction.

### Sputum sampling

We collected spontaneous sputum among all bronchiectasis patients, and induced sputum (3% hypertonic saline) in healthy subjects. Participants forcefully coughed up sputum after gargling mouth with distilled water. We sampled the most purulent portion for quality control. We evaluated the presence/absence of eosinophilia or neutrophilia [25] and stored freezing in − 80 degree freezers for metagenomic sequencing. See further details in Additional file 14.

### Metagenomic sequencing

Bacterial genomic DNA was extracted using Qiagen DNA Mini kit, and homogenized using sterile zirconium/silica beads. Sequencing libraries were generated using NEB Next® Ultra™ DNA Library Prep Kit for Illumina® (New England Biolabs, MA, USA), followed by ultra-deep sequencing using Illumina NovaSeq 6000 (targeted >  = 40G sequences per sample, 2 × 150 bp paired-end sequencing). Two DNA extraction blank controls were sequenced.

Raw reads were processed using the Sunbeam pipeline [26], in which the adaptor sequences were removed by using Cutadapt (v2.5) [6], and quality filtered using Trimmomatic (v0.36) (setting: ILLUMINACLIP: NexteraPE-PE.fa:2:30:10:8:true LEADING:3TRAILING:3 SLIDINGWINDOW:4:15 MINLEN:36) [8]. Low-complexity sequences were filtered using Komplexity [26]. FastQC (v0.11.8) was used for quality check. Next, BWA (v0.7.17) was used to align quality-filtered reads to human reference genome (hg38) [7]. The sequencing depth was 40 G/sample. The average and median non-human read count across all samples was 16,792,475 and 7,852,636, respectively (range: 1,616,520–173,251,576). Taxonomic classification of non-human reads was performed using Kraken2 [9]. Raw data have been deposited in the Genome Sequence Archive in Beijing Institute of Genomics Data Center, under BioProject accession PRJCA013397 (https://ngdc.cncb.ac.cn/gsa-human/browse/HRA003749, No.: HRA003749).

### Quantification of ARGs

Non-human reads were matched against the *Structured Antibiotic Resistance Gene* database (SARG) by using BLASTX [27], as described previously [28–30]. The reads which met BLASTX criteria (alignment length 75 bp, similarity 80%, e-value 10^−7^) were classified according to the SARG hierarchy. ARG abundance was calculated as reads per kilobase of reference sequence per million sample reads (RPKM) [31].

### Statistical analysis

No sample size estimation was performed. Unsupervised cluster analysis was performed using the average linkage method of the hclust function in R stat package. The beta-diversity of microbiome and ARG profiles was presented using *Principal Co-ordinate Analysis* (PCoA), and *ANalysis Of SiMilarities* was used to test the statistical significance between groups. Permutational multivariate analysis of variance using distance matrices analysis (Adonis) was performed using R vegan package. To compare ARG abundance for paired stable-exacerbation samples, paired Wilcoxon rank-sum test was performed. Redundancy analysis was conducted to visualize the impact of clinical characteristics on ARG profiles. Spearman’s correlation was performed for continuous variables and Wilcoxon signed-rank test for categorical variables. Shannon index was calculated to evaluate the alpha diversity of ARGs. Procrustes analysis was performed to determine the agreement between resistome and microbiota compositions with Procrustes function in R vegan package.

For each variable, we performed FDR adjustment for P-values. The FDR-corrected *P* < 0.05 was deemed statistically significant. Analysis was performed using R (version 4.1.0), with the ggplot2 package for visualization. Heat maps and Venn diagram were produced using R with Pheatmap and VennDiagram package, respectively.

## Results

### Baseline characteristics

We enrolled 101 study participants, including 82 bronchiectasis patients and 19 healthy controls. Seventeen patients had paired stable and exacerbation samples, resulting in 118 sputum samples being analyzed. Baseline characteristics are shown in Table 1. 82 Bronchiectasis patients consisted of mostly never-smoked middle-aged adults with predominantly mild-to-moderate bronchiectasis (median BSI: 6.0). 15.8% had co-existing asthma while 7.1% had sputum eosinophilia (eosinophils ≥ 3%). 61.0% and 13.4% had blood eosinophil count greater than 100 and 300 cells per microliters, respectively. Overall, 20% had exposures to different classes of antibiotics within the previous 6 months, with a slightly higher frequency of beta-lactams (22.0% vs. 35.3%) and fluoroquinolones (23.2% vs. 41.2%) in the exacerbation cohort. The clinical characteristics of the exacerbation cohort were comparable with the whole bronchiectasis cohort, with nominally lower lung function, greater disease severity and more frequent antibiotic use in the exacerbation cohort. Furthermore, there were no remarkable differences in the age, gender and smoking between bronchiectasis patients and healthy controls (all *P* > 0.05).

**Table 1:** Baseline characteristics of bronchiectasis patients and healthy controls

Variables	Stable cohort (n = 82)	Exacerbation cohort (n = 17)	Healthy controls (n = 19)
Age (years)	48.2 ± 13.0	50.0 ± 13.4	53.1 ± 11.4
Females no. (%)	44 (53.7%)	8 (47.1%)	7 (36.8%)
Body-mass index (Kg/m^2^)	20.6 ± 3.1	21.0 ± 3.0	24.3 ± 3.4
FEV_1_% predicted	60.7 ± 20.1	52.8 ± 21.0	94.7 ± 12.3
Never-smokers (No. %)	74 (90.2%)	16 (94.1%)	17 (89.5%)
Co-existing asthma (No. %)	13 (15.8%)	1 (5.9%)	NA
Co-existing COPD (No. %)	4 (4.9%)	2 (11.8%)	NA
Exacerbation frequency in the previous year	1.0 (1.0)	1.0 (1.0)	NA
Blood eosinophil > 100/μl (No. %)^a^	50 (61.0%)	10 (58.8%)	NA
Blood eosinophil > 300/μl (No. %)^a^	11 (13.4%)	2 (11.8%)	NA
Sputum eosinophilia (No. %)^a^	2 (7.1%)	0 (0%)	NA
Sputum neutrophilia (No. %)^a^	26 (92.9%)	8 (100.0%)	NA
Modified Reiff score	9.3 ± 3.8	11.4 ± 3.5	NA
Aetiology			
Idiopathic (No. %)	40 (48.7%)	9 (52.9%)	NA
Post-infectious (No. %)	25 (30.5%)	5 (29.4%)	NA
Others (No. %)	17 (20.7%)	3 (17.6%)	NA
Bronchiectasis severity index	6.0 (7.0)	7.9 ± 4.4	NA
Bronchiectasis severity			
Mild (No. %)	30 (36.6%)	4 (23.5%)	NA
Moderate (No. %)	25 (30.5%)	6 (35.3%)	NA
Severe (No. %)	27 (32.9%)	7 (41.2%)	NA
Medications within 6 months			
Inhaled corticosteroids (No. %)	14 (17.1%)	3 (17.6%)	NA
Long-acting beta-agonists (No. %)	15 (18.3%)	1 (5.9%)	NA
Maintenance macrolides (No. %)	11 (13.4%)	2 (11.8%)	NA
Beta-lactams (No. %)	18 (22.0%)	6 (35.3%)	NA
Fluoroquinolone (No. %)	19 (23.2%)	7 (41.2%)	NA

Continuous variables were checked for normality, and expressed as mean ± standard or median (interquartile range) as appropriate. Categorical variables were summarized as count by (percentage)

*FEV*_*1*_  forced expiratory volume in one second, *pred%*  the percentage predicted, *NA*  not applicable, *COPD*  chronic obstructive pulmonary disease

Other aetiologies of bronchiectasis consisted of primary ciliary dyskinesia (n = 7), immunodeficiency (n = 8), gastroesophageal reflux (n = 1), and connective tissue disease (n = 1); other aetiologies of patients who had an exacerbation consisted of primary ciliary dyskinesia (n = 1) and immunodeficiency (n = 1)

Sputum eosinophilia denotes sputum eosinophil count exceeding 2.5% of the differential counts, and sputum neutrophilia denotes sputum neutrophil count exceeding 61.0% of the differential counts

^a^There were a total of 28 patients in the stable cohort and 8 in the exacerbation cohort who had sufficient sputum for cell differentials [25]

The exacerbation column denotes the bronchiectasis patients with an outpatient visit due to having an exacerbation during the longitudinal follow-up

### Overview of airway resistome and correlation with disease severity metrics

**Fig. 1:**
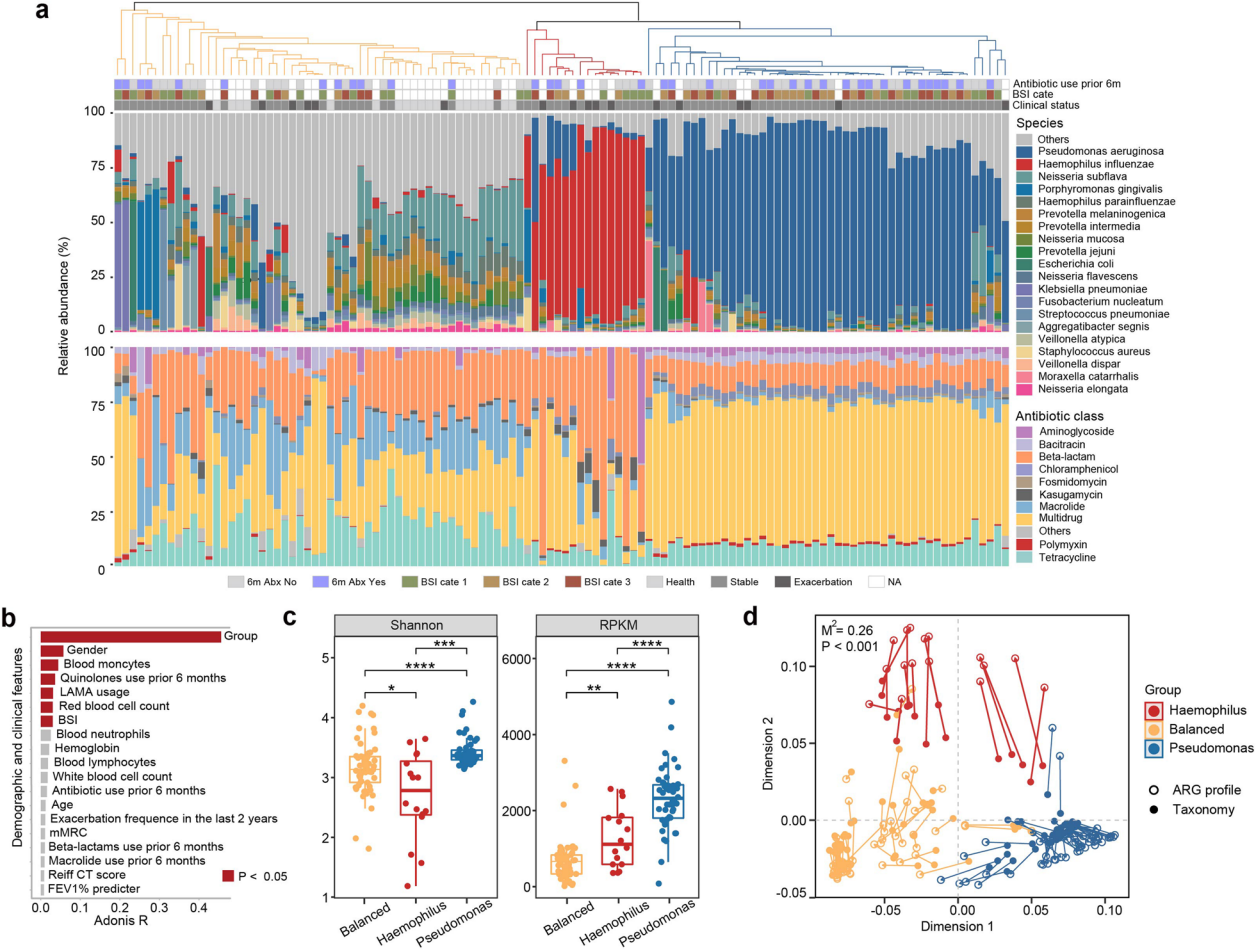
Profiles of antibiotic resistance genes (ARGs) in bronchiectasis patients and healthy controls. **A** A heat map demonstrating the bacterial species-level taxa and ARGs in 118 sputum samples, with unsupervised clustering revealing the *Pseudomonas* predominant, *Haemophilus* predominant, and a balanced microbiome composition subgroup. The upper panel demonstrates the bacterial compositions whereas the lower panel displays the ARG categories. **B** Association between clinical characteristics and the ARG profile. **C** The box and dot plots revealing the ARG diversity and total abundance among the *Pseudomonas*-predominant subgroup (*Pseudomonas* relative abundance: 73.0% ± 20.6%), *Haemophilus*–predominant subgroup (*Haemophilus* relative abundance: 70.8% ± 15.3%) and balanced microbiota subgroup (no single dominant microbe). **D** Correlation between the sputum microbiota and ARG composition. *ARG* antibiotic resistance gene, *LAMA* long-acting muscarinic antagonist, *BSI* bronchiectasis severity index, *mMRC* modified Medical Research Council dyspnea scale, *CT* computed tomography, *FEV*_*1*_ forced expiratory volume in one second, *RPKM* reads per kilobase of gene per million. All P values for the statistical analyses have been corrected with the false discovery rate

Comparison of the DNA extraction blank controls and sputum revealed marked differences in the metagenomic compositions that precluded the contamination by microbiota from the sequencing reagents or procedures (Additional file 1: Fig. S1). Metagenomic sequencing identified 272 bacterial species-level taxa with a relative abundance above 10^–4^ and 307 ARGs in all 118 sputum samples. Both the ARGs and the microbial compositions differed considerably between bronchiectasis patients and healthy controls. Furthermore, unsupervised clustering revealed three subgroups of microbiome (Fig. 1a): *Pseudomonas*-predominant subgroup (11 stable and 5 exacerbation samples, n = 16; *Pseudomonas* relative abundance: 73.0 ± 20.6%), *Haemophilus*-predominant subgroup (42 stable and 6 exacerbation samples, n = 48; *Haemophilus* relative abundance: 70.8 ± 15.3%); a balanced microbiome composition subgroup (29 stable and 6 exacerbation samples, plus samples from 19 healthy individuals, n = 54; no single dominant microbe, Additional file 8: Table S1). Distinct resistome profiles were observed among the three groups (Fig. 1a). ARGs of multi-drug resistance were dominant in the *Pseudomonas*-predominant subgroup, while ARGs of beta-lactam resistance were most highly abundant in *Haemophilus*-predominant subgroup (Additional file 2: Fig. S2). Adonis analysis suggests that the microbiome-based clusters were most strongly correlated with the ARG profile. Other than the microbiome clusters, gender, blood monocytes, quinolone use within the prior 6 months (analyzed as an independent variable instead of confounding variable), long-acting muscarinic antagonist use, and BSI were significantly associated with the ARG profile (Adonis *P* < 0.05, Fig. 1b, Additional file 2: Table S2). An overall balanced resistome profile was observed in the balanced microbiota subgroup. *Pseudomonas*-predominant subgroup had the highest ARG diversity and total abundance, while *Haemophilus*–predominant subgroup and balanced microbiota subgroup were lowest in ARG diversity and total abundance, respectively (Fig. 1c, Additional file 3: Fig. S3). A robust and significant correlation was observed between the microbiota and ARG profiles, as revealed by Procrustes analysis (Fig. 1d, Additional file 4: Fig. S4).

**Fig. 2:**
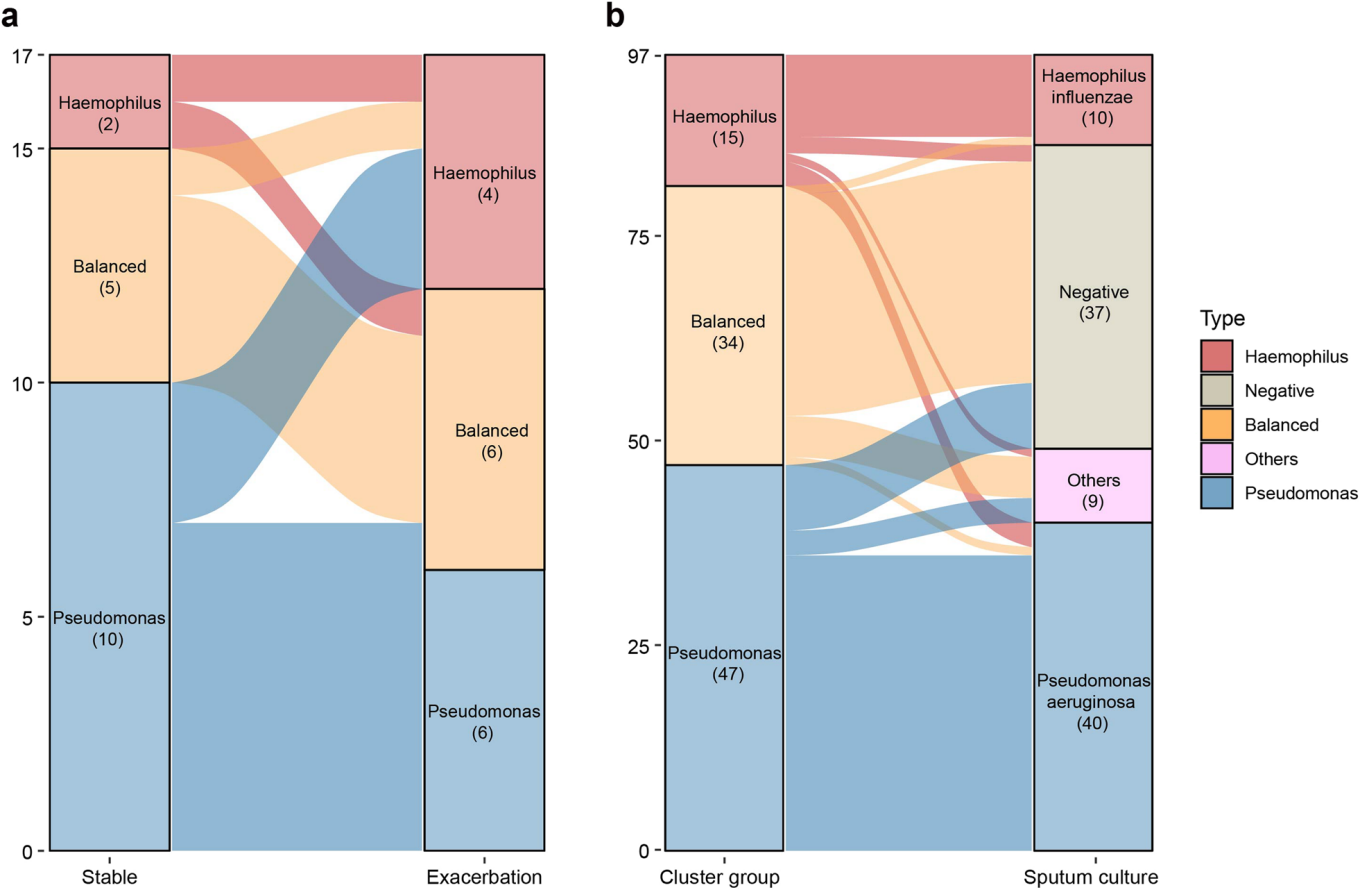
Microbial compositions of bronchiectasis patients when clinically stable and at exacerbation onset. **A** Transition patterns of the microbial subtypes between the paired stable and exacerbation samples. **B** Agreement between the microbial subtypes and the bacterial culture results. The 9 sputum samples that were listed as ‘others’ in culture results are: *Moraxella catarrhalis* (n = 2), *Klebsiella pneumoniae* (n = 4), *Pseudomonas fluorescens* (n = 1), *Staphylococcus aureus* (n = 1), *Streptococcus pneumoniae* (n = 1)

The microbiome clusters was overall comparable between the paired stable and exacerbation samples, with a transition from the *Pseudomonas*-predominant to the *Haemophilus*-predominant subtype in three patients (Fig. 2a). Notably, there was a reduction in the abundance of *Pseudomonas aeruginosa* at exacerbation onset compared with the stable state. The reduction of *Pseudomonas aeruginosa* relative abundance at exacerbation onset was non-significant (FDR = 0.265, paired Wilcoxon test). In addition, the decreased representation of *Pseudomonas*-predominant subgroup at exacerbation onset as compared with that of the stable state was non-significant (*P* = 0.599, Fisher’s exact test). A greater representation of ARGs of beta-lactam resistance over multi-drug resistance was observed during exacerbations (Additional file 5: Fig. S5). The microbial subtypes based on metagenomic sequencing highly concurred with bacterial culture results (Fig. 2b).

We have compared the samples collected prior to and after the COVID-19 outbreak (cut-off: January 2020) with PCoA and ANOSIM, revealing no major differences in the microbial compositions and ARG profiles (Additional file 6: Fig. S6).

### Differential association between resistome and clinical characteristics in *Haemophilus*- and *Pseudomonas*-predominant subtypes

**Fig. 3:**
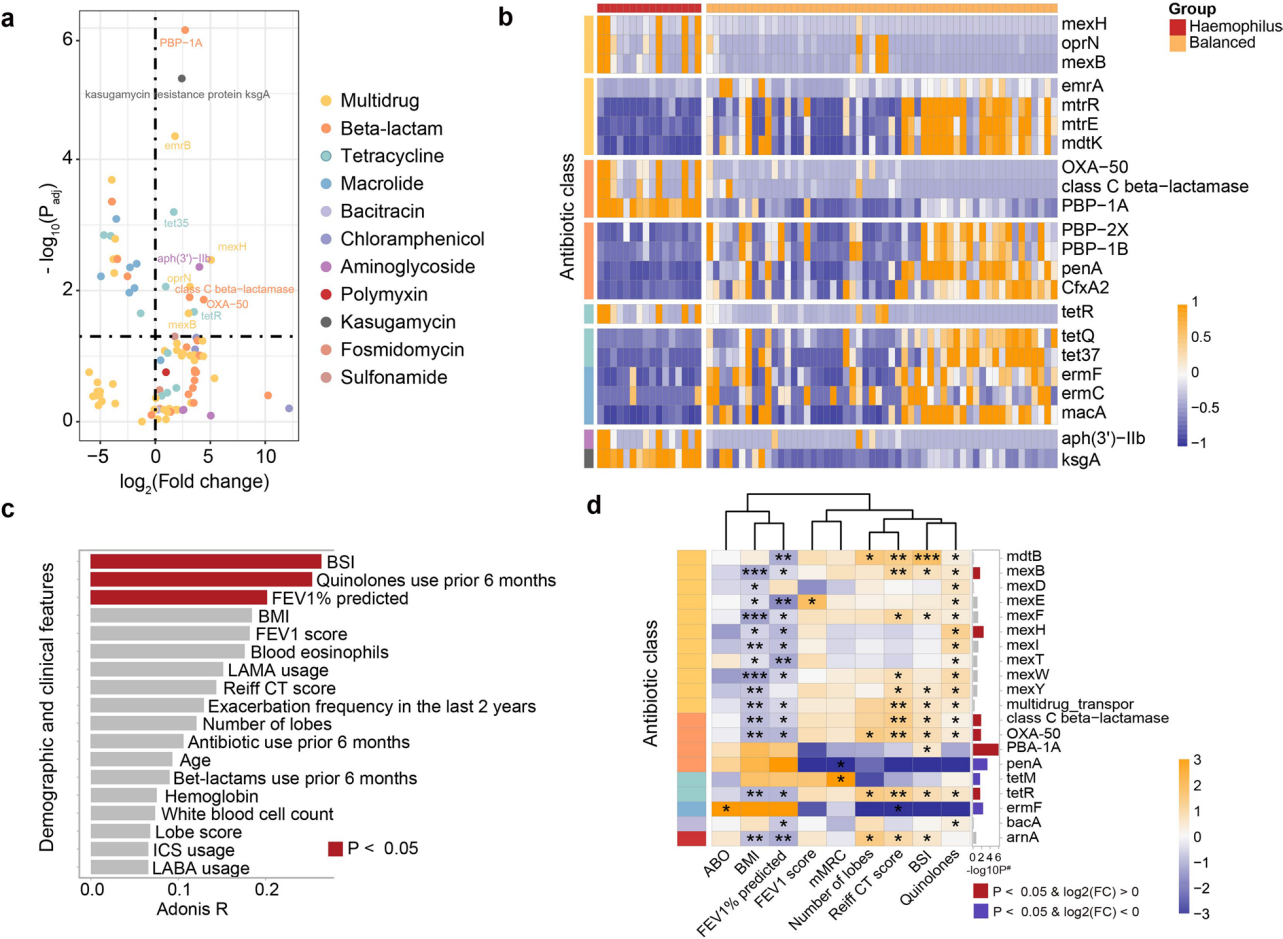
The distribution of antibiotic resistance genes in bronchiectasis patients with *Haemophilus*-predominant microbial profiles when clinically stable. **A** Abundance profiles of the enriched (log2 fold-change > 0) or depleted ARGs (log2 fold-change < 0) in *Haemophilus*-predominant versus balanced microbiota subtypes. Only ARGs with relative abundance greater than 0.005 are shown for display purpose; **B** A heat map demonstrating the profiles of differential ARGs between stable samples within *Haemophilus*-predominant and the balanced microbiota subgroups. Only ARGs with relative abundance greater than 0.005, with absolute fold-change greater than 2.0, and with FDR *P* < 0.05 are shown; **C** Association between the core clinical parameters and the *Haemophilus*-predominant ARG profile; **D** A heat matrix displaying the strength of association between the ARGs and the core clinical metrics. Only ARGs with relative abundance greater than 0.001 are shown for display purpose. *ARG* antibiotic resistance gene, *ABO* asthma-bronchiectasis overlap, *BMI* body-mass index, *BSI* bronchiectasis severity index, *mMRC* modified Medical Research Council dyspnea scale, *CT* computed tomography, *FEV*_*1*_ forced expiratory volume in one second. All P values for the statistical analyses have been corrected with the false discovery rate

Within samples from stable state, we identified differential ARGs in *Haemophilus* and *Pseudomonas*-predominant subtypes, compared with the balanced microbiota subtype. When clinically stable, the abundance of 12 ARGs were significantly enriched and 16 ARGs were depleted in the *Haemophilus*-predominant subtype as compared with the balanced microbiota subtypes (FDR *P* < 0.05, Fig. 3a, Additional file 10: Table S3). *PBP-1A* (beta-lactam), *ksgA* (kasugamycin) and *emrB* (multidrug) were most significantly enriched in *Haemophilus*-predominant subtype (Fig. 3b). BSI was most strongly associated with the *Haemophilus*-predominant ARG profile (Additional file 9: Table S2), followed by quinolone use in the previous 6 months and FEV_1_% predicted (Fig. 3c, Adonis *P* < 0.05). Most ARGs enriched in *Haemophilus*-predominant subtype yielded positive correlations with the BSI, fluoroquinolone use, and modified Reiff score, while they were mostly negatively correlated with the body-mass index and FEV_1_% predicted (Fig. 3d, Additional file 11: Table S4). Specifically, 10 ARGs significantly and positively correlated with BSI, including *PBP-1A* that was most significantly enriched in *Haemophilus*-predominant subtype (Fig. 3d).

**Fig. 4:**
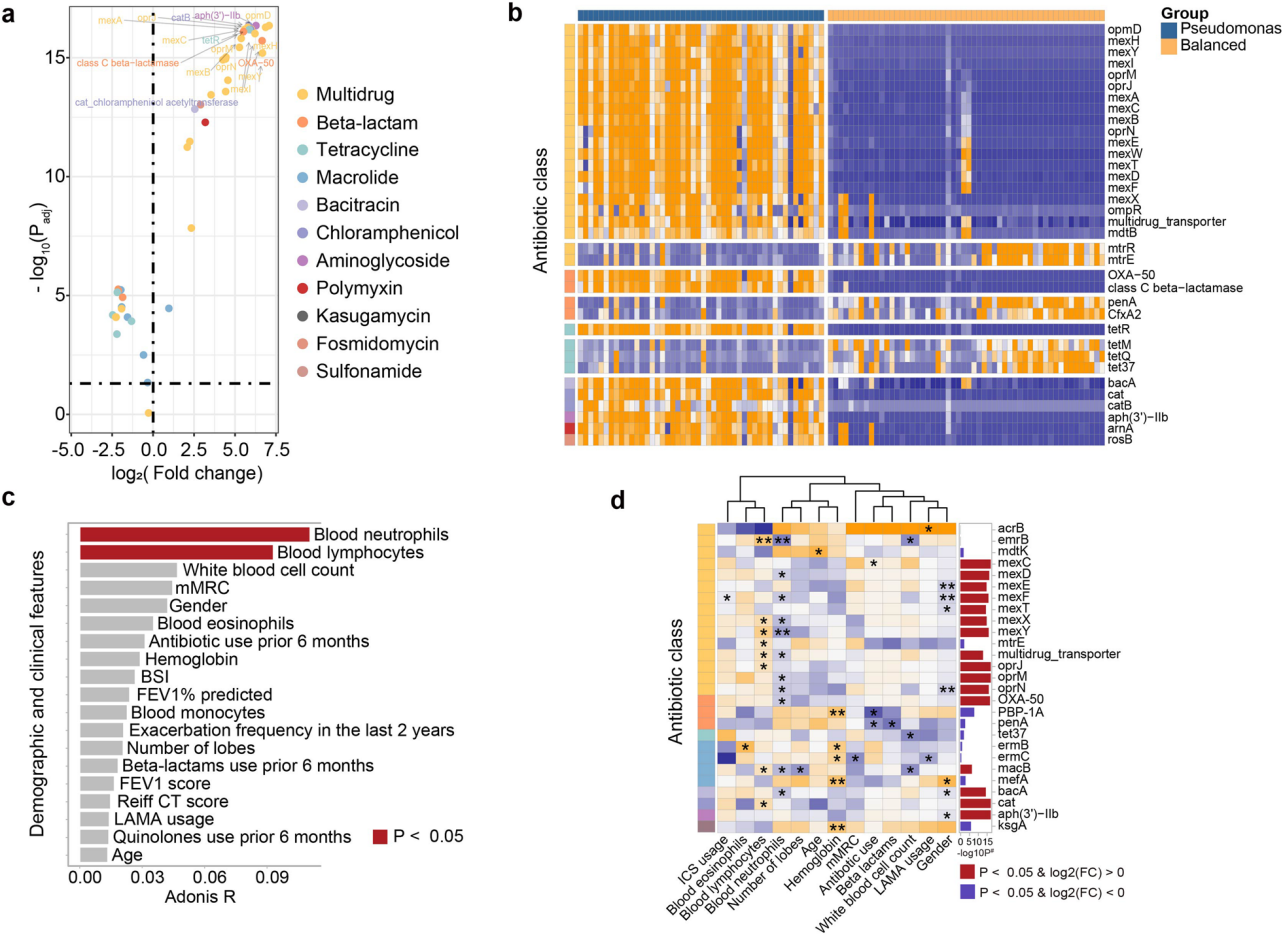
The distribution of antibiotic resistance genes in bronchiectasis patients with *Pseudomonas*-predominant microbial profiles when clinically stable. **A** Abundance profiles of the enriched (log2 fold-change > 0) or depleted ARGs (log2 fold-change < 0) in *Pseudomonas*-predominant versus balanced microbiota subtypes. Only ARGs with relative abundance greater than 0.005 are shown for display purpose; **B** A heat map demonstrating the profiles of differential ARGs between stable samples within *Pseudomonas*-predominant and the balanced microbiota subgroups. Only ARGs with relative abundance greater than 0.005, with absolute fold-change greater than 2.0, and with FDR *P* < 0.05 are shown; **C** Association between the core clinical parameters and the *Pseudomonas*-predominant ARG profile; **D** A heat matrix displaying the strength of association between the ARGs and the core clinical metrics. Only ARGs with relative abundance greater than 0.001 are shown for display purpose. *ARG* antibiotic resistance gene, *ABO* asthma-bronchiectasis overlap, *BMI* body-mass index, *BSI* bronchiectasis severity index, *mMRC* modified Medical Research Council dyspnea scale, *CT* computed tomography, *FEV*_*1*_ forced expiratory volume in one second All P values for the statistical analyses have been corrected with the false discovery rate

Thirty-four and 27 ARGs were significantly enriched or depleted in *Pseudomonas*-predominant versus the balanced microbiota subtype (FDR *P* < 0.05, Fig. 4a, Additional file 10: Table S3), with most enriched ARGs bearing multidrug resistance (Fig. 4a, b). Blood neutrophil and lymphocyte percentages were most strongly associated with the *Pseudomonas*-predominant ARG profile (Fig. 4c, Additional file 9: Table S2). Specifically, they exhibited significant inverse correlations with multiple ARGs of multi-drug resistance, including *emrB*, *mexXY*, and a multi-drug transporter (Fig. 4d, Additional file 11: Table S4). Therefore, there existed differential sets of clinical parameters which were associated with the resistome in *Haemophilus-* and *Pseudomonas*-predominant subtypes in stable bronchiectasis, respectively.

### Validation of associations between resistome and clinical parameters

Finally, we analyzed data in an independent cohort (Singapore, United Kingdom, Italy, Malaysia) [11], with the same analytical procedure and pipeline. Unsupervised clustering similarly revealed three microbiome clusters, with an over-representation of the balanced microbiota subgroup compared with our cohort (the Guangzhou cohort, Additional file 7: Fig. S7). The number of *Klebsiella*-predominant was 2 in our dataset (Fig. 1) and 4 in the validation dataset (Additional file 7: Fig. S7). Due to the small sample size, it was difficult to statistically assess the clinical characteristics for this particular subgroup of patients. Of the 61 and 28 ARGs associated with *Pseudomonas*-predominant and *Haemophilus*-predominant subgroups in the Guangzhou cohort respectively, 52 and 20 ARGs exhibited the same directionality of changes in the validation dataset, with 40 and 6 ARGs being statistically significant (FDR *P* < 0.05). In the validation dataset, the microbiome cluster, macrolides and ICS use were associated with the ARG profile (FDR *P* < 0.10, Additional file 12: Table S5). Macrolides use and age were associated with the ARG profile in *Pseudomonas*-predominant subgroup, while geography, macrolides use, gender and BSI were associated with the ARG profile in *Haemophilus*-predominant subgroup (FDR *P* < 0.25, Additional file 12: Table S5). There were 105, 23 and 71 correlations between ARG and the clinical features that were shared between the two datasets in all samples, *Pseudomonas*-predominant, and *Haemophilus*-predominant subgroups, respectively (FDR *P* < 0.10). Of these figures, 72, 18 and 45 correlations in all samples, *Pseudomonas*-predominant, and *Haemophilus*-predominant subgroups exhibited the same directionality in the validation dataset (Additional file 13: Table S6).

## Discussion

We performed metagenomic shotgun sequencing on spontaneous sputum from bronchiectasis patients, revealing distinct profiles of ARGs associated with three microbial subgroups. The ARG profile was associated with multiple clinical metrics. The *PBP-1A*, *ksgA* and *emrB* genes were enriched in *Haemophilus*-predominant subgroup, whereas the *emrB*, *mexXY* genes were depleted in *Pseudomonas*-predominant subgroup. These findings could be partly validated (~ 68.6% of the ARG-clinical correlations) in an independent international cohort [11, 32, 33], indicating the generalizability to other study populations.

Concerns have been raised regarding the accumulation of ARGs in bronchiectasis. By comparison with healthy controls, Mac Aogain and colleagues documented the prevalent ARGs associated with fluoroquinolone, beta-lactam and tetracycline which were independent of prior antibiotic exposures and the clinical status of CRDs [11]. Congruent with these observations, our study has further identified the ARGs of beta-lactam resistance in the *Haemophilus*-predominant subgroup, multi-drug resistance in the *Pseudomonas*-predominant subgroup. The two studies, however, differed in patient populations (three CRDs vs. Bronchiectasis) and study objective (profiling resistome/microbiome and the inhaler microenvironment vs. profiling resistome according to microbiome and clinical characteristics). Our findings pertaining to ARGs associated with *Pseudomonas* spp. were clinically relevant. Sputum microbiota with predominance of *Pseudomonas* spp. has been associated with a low microbial diversity and greater severity of bronchiectasis [34]. Other studies [7, 8, 35] have reported the predominance of *Pseudomonas* associated with multi-drug resistance in cystic fibrosis (CF). Other cellular mechanisms (e.g., active reflux, porin alterations) might have also contributed to the acquired resistance to antibiotics for *Pseudomonas* spp. [36]. Furthermore, the *ampC* (encoding beta-lactamase) and *ftsl* gene (encoding penicillin-binding protein 3) mutations were frequently detected in *P. aeruginosa* [27], and both *fusA1* and *pmrB* mutations conferred polymyxin resistance in CF [9].

The *Haemophilus*-dominant taxa was common among bronchiectasis patients [1, 37]. As per international guidelines [38], beta-lactams are first-line antibiotics for bronchiectasis patients isolated with *H. influenzae* at exacerbation onset. The ARGs including *bla*_*TEM-1D*_ (ampicillin resistance) and *tetB* genes (tetracycline resistance) have also been detected, despite the lower prevalence (6.1%) in patients with other airway diseases [39]. Apart from beta-lactam resistance, a rarer but high-level ciprofloxacin-monoresistant *H. influenzae* (mainly involving *gyrA*, *parC* and *parE* mutations) isolate has been identified [40], indicating the mutational capacity of ARGs in *H. influenzae*. Consistent with findings from COPD [14], ARGs were largely determined by the microbial compositions. The balanced subgroup was characterized by the intermediate ARG profile, which has also been shaped by other microbiota taxa. Antibiotic resistance associated with oral commensals such as *Prevotella* spp., which was generally resistant to amoxicillin (96%) and occasionally resistant to multiple drugs (10%), has been detected despite the less prominent (24%) resistance to moxifloxacin [41].

The ARG profiles in bronchiectasis did not differ significantly when comparing clinically stable samples and those at exacerbation onset, which mirrored the findings from COPD [14]. The microbial compositions during clinical stability and exacerbation mostly remained stable. In COPD, antibiotics therapy targeting at ameliorating exacerbations did not enrich any particular microbial taxa or ARG [14]. Hence, the ARG profiles may be independent of the clinical status.

We identified reproducible correlations between certain ARG profiles and the clinical metrics of bronchiectasis. The cardinal factors shaping ARGs consisted of sex, blood monocytes, quinolone or LAMA use, and the BSI. Blood neutrophil and lymphocyte percentages correlated strongly with the *Pseudomonas*-dominant ARG profile. The association between blood inflammatory cells and ARG profiles in bronchiectasis merits further comments. A cross-sectional study reported a marked difference in ARG profiles between neutrophil-dominant COPD (dominated by multidrug resistance genes related to *Ralstonia* and *Pseudomonas*) and eosinophil-dominant COPD (macrolide-high resistome predominantly associated with *Campylobacter* and *Aggregatibacter*) [42]. Concurrent profiling of the metabolic pathways and inflammatory biomarkers will help to shed mechanistic insights into our observations. The precise mechanisms underlying the association between LAMA use and ARG profiles are not entirely clear, whether this relates to the anti-inflamamtory actions of LAMA that could shape the microbial compositions merits further investigation.

Our study has profiled the ARGs abundance, in a well characterized cohort of bronchiectasis patients, by linking ARG profiles to disease severity, inflammatory subtypes and prior antibiotic exposures. Metagenomic sequencing has minimized the risk of biased analysis (e.g., polymerase chain reactions). Profiling of the dominant microbiota taxa and key clinical metrics (e.g. inflammatory subtypes, disease severity) will enable a better understanding of the factors associated with microbial resistance, for instance, revealing the patient subgroups most likely harboring extensive ARGs.

However, our findings was limited by the inability to evaluate dynamic changes (e.g. repeated assessments when clinically stable) within an individual. Direct comparison of spontaneous sputum in bronchiectasis patients vs. induced sputum in healthy controls has been problematic; however, obtaining bronchoalveolar lavage would be constrained by the invasiveness and cost, reducing the feasibility with much larger sample sizes. Despite the substantial difference in microbiota compositions detected with 16 s rRNA sequencing between induced sputum and spotaneous sputum in patients with COPD [43], data regarding the comparison with metagenomic sequencing are lacking. Although not directly comparable due to the notable differences in specimen processing and sequencing techniques and demographic characteristics, our principal findings could be validated in a multinational cohort, which has partly circumvented the single-country study design. The overal cohort is relatively mild with a mild-moderate BSI score and a low median exacerbation frequency the previous year, and hence extrapolation of our conclusion should be taken cautiously. We employed a previously validated read-based approach to profile ARGs, however, future validation with assembly-based algorithms would be needed. We have standardized the relative abundance of ARGs based on the sequencing throughput which allowed for direct comparisons among different samples, however, the batch effects related to nucleic acid extract and sequencing cannot be fully addressed. There was no control for *Pseudomonas* ARGs for healthy controls; however, the lack of *Pseudomonas*- or *Haemophilus*-dominant ARG subtype precluded our further analysis. This might be circumvented by the comparison with ARGs in other CRDs. We noted the distinct resistome in different microbial-dominant subgroups, albeit the sample size for the *Pseudomonas*-dominant subgroup was insufficient to fully confirm our findings. Finally, although ARGs were associated with clinical severity and macrolide use, the prognostic implications are not entirely clear. Integration of ARG profiling with other measures (e.g. metabolome) may enable further clues as to how ARGs interact with host immune response particularly during exacerbation dynamics.

In conclusion, we have unraveled the differentially abundant ARG profiles enriched or depleted in bronchiectasis patients with distinct predominant microbial species. Future studies should enable the scientific community to understand longitudinal evolution/acquisition, and whether this is related to environmental niche, the effect from antibiotics and the impact of broad spectrum antibiotics in the context of exacerbation.

### Supplementary Information

**Additional file 1: Figure S1.** Comparison of the microbial compositions between two DNA extraction blank controls and the quality-controlled sputum from patients with bronchiectasis. Shown in the left side of the left and right panels of each figure are the microbial compositions from two DNA extraction blank controls. The samples denote the sputum derived from patients with bronchiectasis. There exist notable differences in the DNA extraction blank controls and the sputum samples, particularly with regard to the dominant microbial taxa identified in patients with bronchiectasis, precluding the major contamination from the sequencing reagents or procedures.

**Additional file 2: Figure S2.** Comparison of the microbial compositions and ARGs when stratified by the predominant species in patients with bronchiectasis when clinically stable. A Microbial profiles of bronchiectasis patients stratified by the dominant microbial species (*Haemophilus*, *Pseudomonas*, and others); B Profiles of ARGs bronchiectasis patients stratified by the dominant microbial species (*Haemophilus*, *Pseudomonas*, and others); C Principal coordinate analysis demonstrating the distribution of microbial compositions associated with the *Pseudomonas*-, *Haemophilus*-predominant subgroup and the balanced microbial subgroup; D Principal coordinate analysis demonstrating the distribution of ARGs associated with the *Pseudomonas*-, *Haemophilus*-predominant subgroup and the balanced microbial subgroup. ARG: antibiotic resistance gene

**Additional file 3: Figure S3.** The spectra of antibiotic resistance genes among bronchiectasis patients with different disease severity and healthy controls. A Box and dot plot comparing the number of ARGs among the *Pseudomonas*-predominant, *Haemophilus*-predominant, and a balanced microbiome composition subgroup (other); B The Venn diagram demonstrating the overlap and unique ARGs among bronchiectasis patients with different microbial profiles; C Stack bar chart demonstrating the distribution of ARGs among bronchiectasis patients with different microbial profiles based on the specific categories of antibiotics. ARG: antibiotic resistance gene

**Additional file 4: Figure S4.** Correlation between the microbial compositions and the ARG profiles in bronchiectasis patients when clinically stable. A Overall correlation analysis of the three distinct microbial subgroups; B Correlation analysis of the *Haemophilus*-predominant subgroup; C Correlation analysis of the *Pseudomonas*-predominant subgroup; D Correlation analysis of the balanced microbial subgroup (other). *The Pseudomonas*-predominant subgroup was characterized by the *Pseudomonas* relative abundance of 73.0% ± 20.6%, the *Haemophilus*–predominant subgroup by the *Haemophils* relative abundance of 70.8% ± 15.3%, and the balanced microbiota subgroup by no single dominant microbe.

**Additional file 5: Figure S5.** Representation of ARGs of beta-lactam resistance over multi-drug resistance in bronchiectasis patients when clinically stable and at onset of exacerbations.

**Additional file 6: Figure S6.** Comparison of the microbial compositions and ARG profiles between the samples collected prior to and after the COVID-19 outbreak. Shown are the results performed with the principal component analysis and Anosim model.

**Additional file 7: Figure S7.** The sputum resistome in bronchiectasis patients from the Guangzhou cohort and the external validation cohort. Unsupervised clustering revealed three microbiome clusters in the validation dataset. However, there is an over-representation of the balanced microbiota subgroup in the multinational cohort as compared with the Guangzhou cohort.

**Additional file 8: Table S1.** The baseline (stable state) clinical characteristics for individuals classified as *Haemophilus*-predominant, *Pseudomonas*-predominant and balanced microbiome subtypes.

**Additional file 9: Table S2.** The Adonis associations between clinical parameters and the ARG profiles in our dataset, among all stable samples and within the subgroup of *Haemophilus*-predominant and *Pseudomonas*-predominant respectively.

**Additional file 10: Table S3.** The differentially abundant ARGs in comparisons of the *Haemophilus*-predominant and *Pseudomonas*-predominant subgroups versus the balanced microbiota subgroup. Also shown are their statistics in the Mac Aogain et al. dataset.

**Additional file 11: Table S4.** The association between clinical parameters and individual ARGs among all stable samples and within the subgroup of *Haemophilus*-predominant and *Pseudomonas*-predominant respectively.

**Additional file 12: Table S5.** The Adonis associations between clinical parameters and the ARG profiles in the Mac Aogain et al. dataset, among all stable samples and within the subgroup of *Haemophilus*-predominant and *Pseudomonas*-predominant respectively.

**Additional file 13: Table S6.** The associations between the ARGs and clinical parameters that are shared by both our dataset and the Mac Aogain et al. dataset.

**Additional file 14.** A word document detailing additional methods, results, and figure and table legends for the Additional files.

## Data Availability

The data could be shared after publication upon request for use via communication with the corresponding authors.

## Abbreviations

95% CI: 95% Confidence interval
AUC: Area under curve
BSI: Bronchiectasis severity index
COPD: Chronic obstructive pulmonary disease
CRDs: Chronic respiratory diseases
FEV_1_ pred%: The percentage predicted of forced expiratory volume in one second
PCoA: Principal Co-ordinates Analysis
ANOSIM: ANalysis Of SiMilarities
Adonis: Permutational multivariate analysis of variance using distance matrices analysis
RDA: ReDundancy Analysis
FDR: False discovery rate
RPKM: Reference sequence per million sample reads

## References

[1] Dicker AJ, Lonergan M, Keir HR, Smith AH, Pollock J, Finch S, Cassidy AJ, Huang JTJ, Chalmers JD (2021). The sputum microbiome and clinical outcomes in patients with bronchiectasis: a prospective observational study. Lancet Respir Med.

[2] Wang Z, Singh R, Miller BE, Tal-Singer R, Van Horn S, Tomsho L, Mackay A, Allinson JP, Webb AJ, Brookes AJ, George LM, Barker B, Kolsum U, Donnelly LE, Belchamber K, Barnes PJ, Singh D, Brightling CE, Donaldson GC, Wedzicha JA, Brown JR (2018). Copdmap. Sputum microbiome temporal variability and dysbiosis in chronic obstructive pulmonary disease exacerbations: an analysis of the COPDMAP study. Thorax.

[3] Abdel-Aziz MI, Brinkman P, Vijverberg SJH, Neerincx AH, Riley JH, Bates S, Hashimoto S, Kermani NZ, Chung KF, Djukanovic R, Dahl CNSE, Adcock IM, Howarth PH, Sterk PJ, Kraneveld AD, Maitland-van der Zee AH (2021). Sputum microbiome profiles identify severe asthma phenotypes of relative stability at 12 to 18 months. J Allergy Clin Immunol.

[4] Huffnagle GB, Dickson RP, Lukacs NW (2017). The respiratory tract microbiome and lung inflammation: a two-way street. Mucosal Immunol.

[5] Wang Z, Locantore N, Haldar K, Ramsheh MY, Beech AS, Ma W, Brown JR, Tal-Singer R, Barer MR, Bafadhel M, Donaldson GC, Wedzicha JA, Singh D, Wilkinson TMA, Miller BE, Brightling CE (2021). Inflammatory endotype-associated airway microbiome in chronic obstructive pulmonary disease clinical stability and exacerbations: a multicohort longitudinal analysis. Am J Respir Crit Care Med.

[6] Sherrard LJ, Tunney MM, Elborn JS (2014). Antimicrobial resistance in the respiratory microbiota of people with cystic fibrosis. Lancet.

[7] López-Causapé C, Oliver A (2017). Insights into the evolution of the mutational resistome of *Pseudomonas aeruginosa* in cystic fibrosis. Future Microbiol.

[8] López-Causapé C, Sommer LM, Cabot G, Rubio R, Ocampo-Sosa AA, Johansen HK, Figuerola J, Cantón R, Kidd TJ, Molin S, Oliver A (2017). Evolution of the *Pseudomonas aeruginosa* mutational resistome in an international cystic fibrosis clone. Sci Rep.

[9] López-Causapé C, Rubio R, Cabot G, Oliver A (2018). Evolution of the *Pseudomonas aeruginosa* aminoglycoside mutational resistome in vitro and in the cystic fibrosis setting. Antimicrob Agents Chemother.

[10] Drevinek P, Canton R, Johansen HK, Hoffman L, Coenye T, Burgel PR, Davies JC (2022). New concepts in antimicrobial resistance in cystic fibrosis respiratory infections. J Cyst Fibros.

[11] Mac Aogáin M, Lau KJX, Cai Z, Kumar Narayana J, Purbojati RW, Drautz-Moses DI, Gaultier NE, Jaggi TK, Tiew PY, Ong TH, Siyue Koh M, Lim Yick Hou A, Abisheganaden JA, Tsaneva-Atanasova K, Schuster SC, Chotirmall SH (2020). Metagenomics reveals a core macrolide resistome related to microbiota in chronic respiratory disease. Am J Respir Crit Care Med.

[12] Torrens G, van der Schalk TE, Cortes-Lara S, Timbermont L, Del Barrio-Tofiño E, Xavier BB, Zamorano L, Lammens C, Ali O, Ruzin A, Goossens H, Kumar-Singh S, Kluytmans J, Paling F, MacLean RC, Köhler T, López-Causapé C, Malhotra-Kumar S, Oliver A (2022). Susceptibility profiles and resistance genomics of Pseudomonas aeruginosa isolates from European ICUs participating in the ASPIRE-ICU trial. J Antimicrob Chemother.

[13] Zhang L, Forst CV, Gordon A, Gussin G, Geber AB, Fernandez PJ, Ding T, Lashua L, Wang M, Balmaseda A, Bonneau R, Zhang B, Ghedin E (2020). Characterization of antibiotic resistance and host-microbiome interactions in the human upper respiratory tract during influenza infection. Microbiome.

[14] Ramsheh MY, Haldar K, Bafadhel M, George L, Free RC, John C, Reeve NF, Ziegler-Heitbrock L, Gut I, Singh D, Mistry V, Tobin MD, Oggioni MR, Brightling C, Barer MR (2020). Resistome analyses of sputum from COPD and healthy subjects reveals bacterial load-related prevalence of target genes. Thorax.

[15] Mac Aogáin M, Lau KJX, Cai Z, Kumar Narayana J, Purbojati RW, Drautz-Moses DI, Gaultier NE, Jaggi TK, Tiew PY, Ong TH, Siyue Koh M, Lim Yick Hou A, Abisheganaden JA, Tsaneva-Atanasova K, Schuster SC, Chotirmall SH (2020). Metagenomics reveals a core macrolide resistome related to microbiota in chronic respiratory disease. Am J Respir Crit Care Med.

[16] Pailhoriès H, Herrmann JL, Velo-Suarez L, Lamoureux C, Beauruelle C, Burgel PR, Héry-Arnaud G (2022). Antibiotic resistance in chronic respiratory diseases: from susceptibility testing to the resistome. Eur Respir Rev.

[17] Rogers GB, Bruce KD, Martin ML, Burr LD, Serisier DJ (2014). The effect of long-term macrolide treatment on respiratory microbiota composition in non-cystic fibrosis bronchiectasis: an analysis from the randomised, double-blind, placebo-controlled BLESS trial. Lancet Respir Med.

[18] Zhao Q, Guo W, Luo H, Xing C, Wang H, Liu B, Si Q, Ren N (2021). Deciphering the transfers of antibiotic resistance genes under antibiotic exposure conditions: driven by functional modules and bacterial community. Water Res.

[19] Samreen AI, Malak HA, Abulreesh HH (2021). Environmental antimicrobial resistance and its drivers: a potential threat to public health. J Glob Antimicrob Resist..

[20] Boyton RJ, Altmann DM (2016). Bronchiectasis: current concepts in pathogenesis, immunology, and microbiology. Annu Rev Pathol.

[21] Hill AT, Haworth CS, Aliberti S, Barker A, Blasi F, Boersma W, Chalmers JD, De Soyza A, Dimakou K, Elborn JS, Feldman C, Flume P, Goeminne PC, Loebinger MR, Menendez R, Morgan L, Murris M, Polverino E, Quittner A, Ringshausen FC, Tino G, Torres A, Vendrell M, Welte T, Wilson R, Wong C, O'Donnell A, Aksamit T (2017). Pulmonary exacerbation in adults with bronchiectasis: a consensus definition for clinical research. Eur Respir J.

[22] Chalmers JD, Goeminne P, Aliberti S, McDonnell MJ, Lonni S, Davidson J, Poppelwell L, Salih W, Pesci A, Dupont LJ, Fardon TC, De Soyza A, Hill AT (2014). The bronchiectasis severity index. An international derivation and validation study. Am J Respir Crit Care Med.

[23] Global Initiatives for Asthma. 2021.

[24] Global Initiatives for Obstructive Lung Disease. 2021.

[25] Brooks CR, van Dalen CJ, Zacharasiewicz A, Simpson JL, Harper JL, Le Gros G, Gibson PG, Pearce N, Douwes J (2016). Absence of airway inflammation in a large proportion of adolescents with asthma. Respirology.

[26] Rolain JM, Fancello L, Desnues C, Raoult D (2011). Bacteriophages as vehicles of the resistome in cystic fibrosis. J Antimicrob Chemother.

[27] Colque CA, Albarracín Orio AG, Feliziani S, Marvig RL, Tobares AR, Johansen HK, Molin S, Smania AM (2020). Hypermutator *Pseudomonas aeruginosa* exploits multiple genetic pathways to develop multidrug resistance during long-term infections in the airways of cystic fibrosis patients. Antimicrob Agents Chemother.

[28] Pettigrew MM, Kwon J, Gent JF, Kong Y, Wade M, Williams DJ, Creech CB, Evans S, Pan Q, Walter EB, Martin JM, Gerber JS, Newland JG, Hofto ME, Staat MA, Fowler VG, Chambers HF, Huskins WC (2022). Comparison of the respiratory resistomes and microbiota in children receiving short versus standard course treatment for community-acquired pneumonia. MBio.

[29] Montassier E, Valdés-Mas R, Batard E, Zmora N, Dori-Bachash M, Suez J, Elinav E (2021). Probiotics impact the antibiotic resistance gene reservoir along the human GI tract in a person-specific and antibiotic-dependent manner. Nat Microbiol.

[30] Yan Z, Chen B, Yang Y, Yi X, Wei M, Ecklu-Mensah G, Buschmann MM, Liu H, Gao J, Liang W, Liu X, Yang J, Ma W, Liang Z, Wang F, Chen D, Wang L, Shi W, Stampfli MR, Li P, Gong S, Chen X, Shu W, El-Omar EM, Gilbert JA, Blaser MJ, Zhou H, Chen R, Wang Z (2022). Multi-omics analyses of airway host-microbe interactions in chronic obstructive pulmonary disease identify potential therapeutic interventions. Nat Microbiol.

[31] Allemann A, Kraemer JG, Korten I, Ramsey K, Casaulta C, Wüthrich D, Ramette A, Endimiani A, Latzin P, Hilty M (2019). Nasal resistome development in infants with cystic fibrosis in the first year of life. Front Microbiol.

[32] Li L, Mac Aogáin M, Xu T, Jaggi TK, Chan LLY, Qu J, Wei L, Liao S, Cheng HS, Keir HR, Dicker AJ, Tan KS, De Yun W, Koh MS, Ong TH, Lim AYH, Abisheganaden JA, Low TB, Hassan TM, Long X, Wark PAB, Oliver B, Drautz-Moses DI, Schuster SC, Tan NS, Fang M, Chalmers JD, Chotirmall SH (2022). Neisseria species as pathobionts in bronchiectasis. Cell Host Microbe.

[33] Mac Aogáin M, Narayana JK, Tiew PY, Ali N, Yong VFL, Jaggi TK, Lim AYH, Keir HR, Dicker AJ, Thng KX, Tsang A, Ivan FX, Poh ME, Oriano M, Aliberti S, Blasi F, Low TB, Ong TH, Oliver B, Giam YH, Tee A, Koh MS, Abisheganaden JA, Tsaneva-Atanasova K, Chalmers JD, Chotirmall SH (2021). Integrative microbiomics in bronchiectasis exacerbations. Nat Med.

[34] Guan WJ, Yuan JJ, Li HM, Gao YH, Huang Y, Chen CL, Chen RC, Zhong NS (2018). Proteobacteria community compositions correlate with bronchiectasis severity. Int J Tuberc Lung Dis.

[35] Sun E, Gill EE, Falsafi R, Yeung A, Liu S, Hancock REW (2018). Broad-spectrum adaptive antibiotic resistance associated with *Pseudomonas aeruginosa* mucin-dependent surfing motility. Antimicrob Agents Chemother.

[36] Chalhoub H, Sáenz Y, Rodriguez-Villalobos H, Denis O, Kahl BC, Tulkens PM, Van Bambeke F (2016). High-level resistance to meropenem in clinical isolates of *Pseudomonas aeruginosa* in the absence of carbapenemases: role of active efflux and porin alterations. Int J Antimicrob Agents.

[37] Guan WJ, Yuan JJ, Li HM, Gao YH, Chen CL, Huang Y, Chen RC, Zhong NS (2018). Altered community compositions of proteobacteria in adults with bronchiectasis. Int J Chron Obstruct Pulmon Dis.

[38] Polverino E, Goeminne PC, McDonnell MJ, Aliberti S, Marshall SE, Loebinger MR, Murris M, Cantón R, Torres A, Dimakou K, De Soyza A, Hill AT, Haworth CS, Vendrell M, Ringshausen FC, Subotic D, Wilson R, Vilaró J, Stallberg B, Welte T, Rohde G, Blasi F, Elborn S, Almagro M, Timothy A, Ruddy T, Tonia T, Rigau D, Chalmers JD (2017). European Respiratory Society guidelines for the management of adult bronchiectasis. Eur Respir J.

[39] Diricks M, Kohl TA, Käding N, Leshchinskiy V, Hauswaldt S, Jiménez Vázquez O, Utpatel C, Niemann S, Rupp J, Merker M (2022). Whole genome sequencing-based classification of human-related Haemophilus species and detection of antimicrobial resistance genes. Genome Med.

[40] Fuursted K, Hartmeyer GN, Stegger M, Andersen PS, Justesen US (2016). Molecular characterisation of the clonal emergence of high-level ciprofloxacin-monoresistant Haemophilus influenzae in the Region of Southern Denmark. J Glob Antimicrob Resist.

[41] Lamoureux C, Guilloux CA, Courteboeuf E, Gouriou S, Beauruelle C, Héry-Arnaud G (2021). Prevotella melaninogenica, a sentinel species of antibiotic resistance in cystic fibrosis respiratory niche?. Microorganisms.

[42] Yi X, Li Y, Liu H, Liu X, Yang J, Gao J, Yang Y, Liang Z, Wang F, Chen D, Wang L, Shi W, Lam DCL, Stampfli MR, Jones PW, Chen R, Wang Z (2022). Inflammatory endotype-associated airway resistome in chronic obstructive pulmonary disease. Microbiol Spectr.

[43] Tangedal S, Aanerud M, Gronseth R, Drengenes C, Wiker HG, Bakke PS, Eagen TM (2017). Comparing microbiota profiles in induced and spontaneous sputum samples in COPD patients. Respir Res.

