# Supplementary figures and images for: Differential airway resistome and its correlations with clinical characteristics in Haemophilus- or Pseudomonas-predominant microbial subtypes of bronchiectasis
Source: Respir Res. 2023 Nov 2;24:264. doi: 10.1186/s12931-023-02562-8 (PMC10623730; doi:10.1186/s12931-023-02562-8)

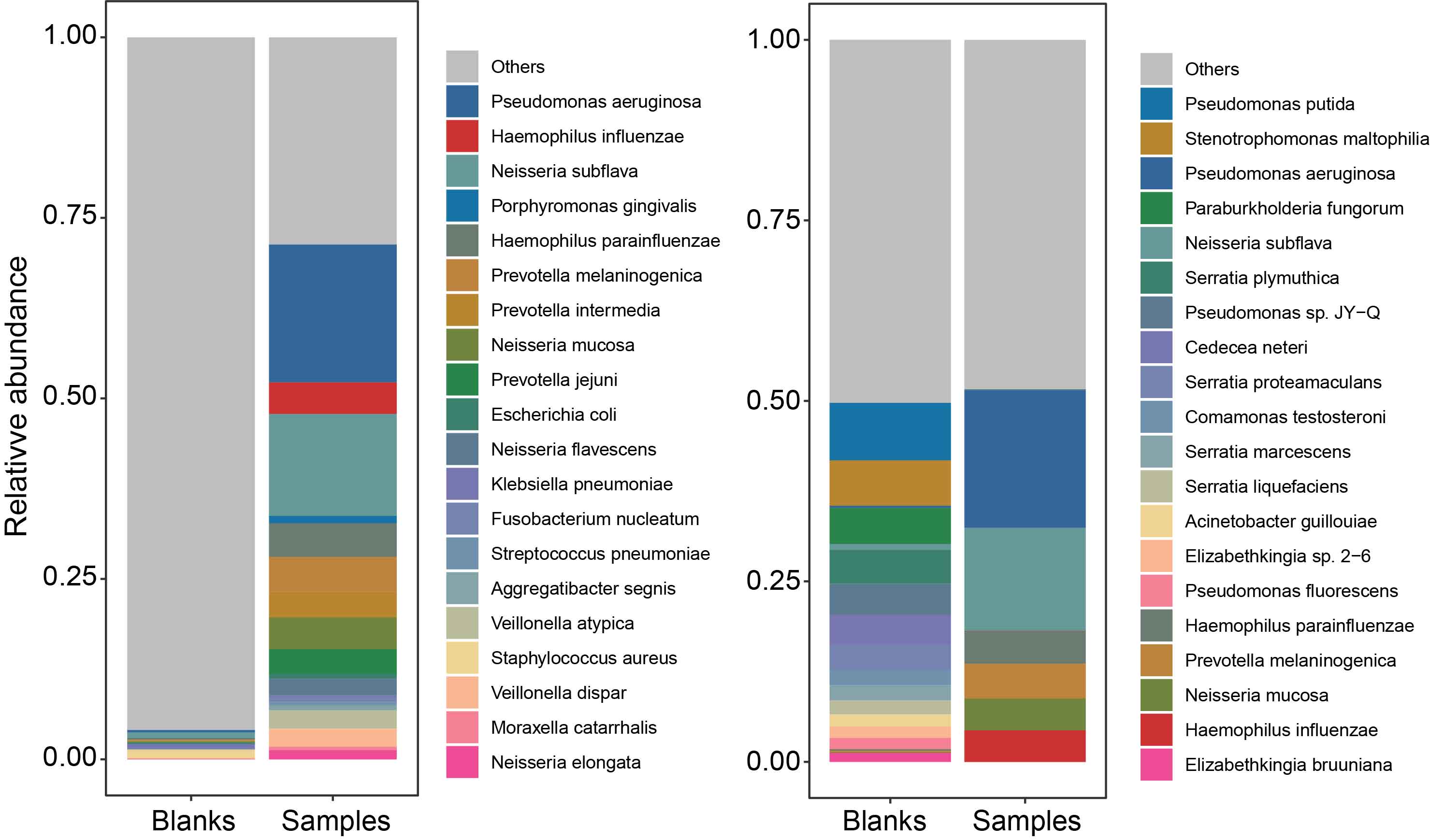

Supplement: Supplementary file 1 — Additional file 1: Figure S1. Comparison of the microbial compositions between two DNA extraction blank controls and the quality-controlled sputum from patients with bronchiectasis. Shown in the left side of the left and right panels of each figure are the microbial compositions from two DNA extraction blank controls. The samples denote the sputum derived from patients with bronchiectasis. There exist notable differences in the DNA extraction blank controls and the sputum samples, particularly with regard to the dominant microbial taxa identified in patients with bronchiectasis, precluding the major contamination from the sequencing reagents or procedures. [file 12931_2023_2562_MOESM1_ESM.jpg]

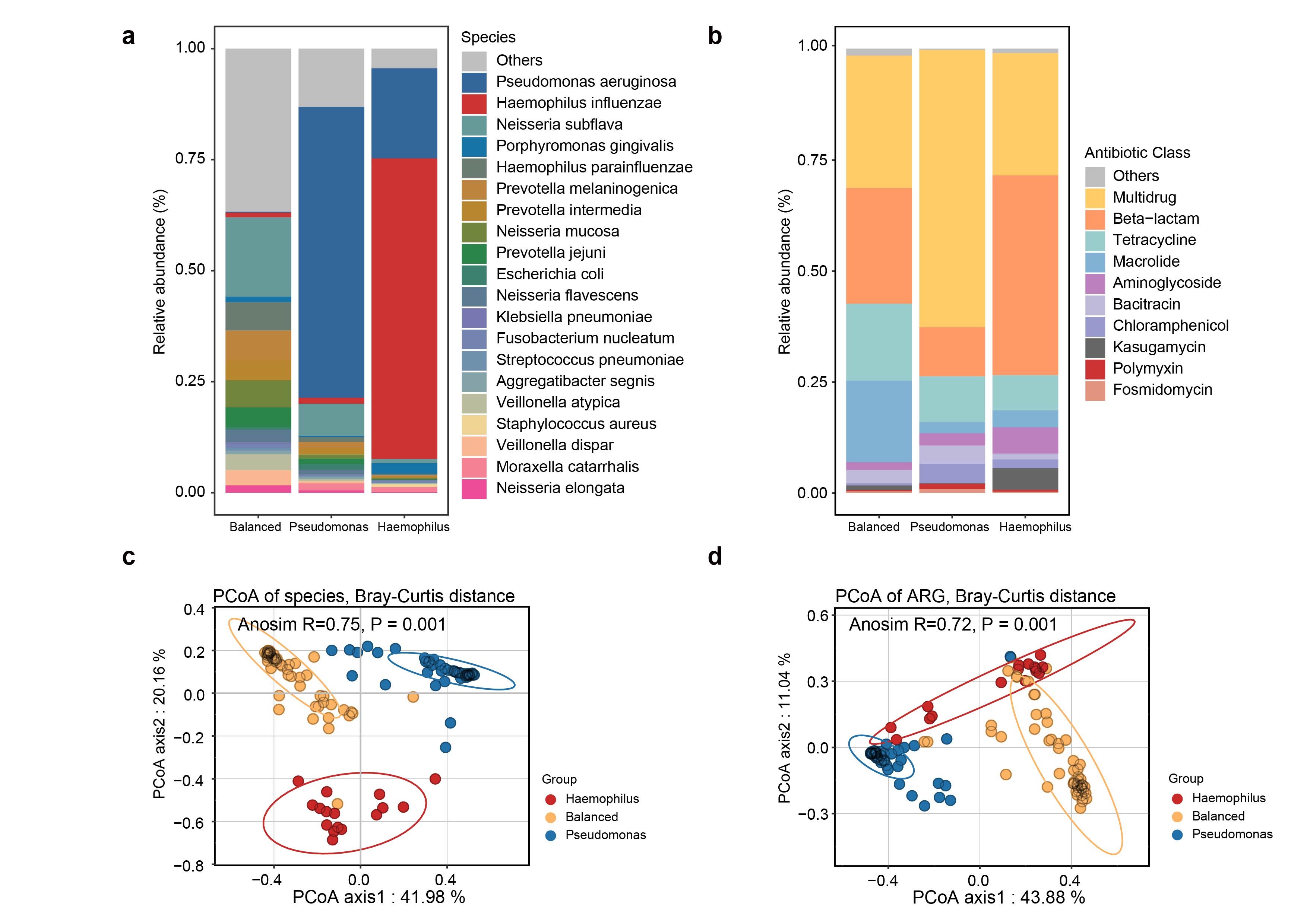

Supplement: Supplementary file 2 — Additional file 2: Figure S2. Comparison of the microbial compositions and ARGs when stratified by the predominant species in patients with bronchiectasis when clinically stable. A Microbial profiles of bronchiectasis patients stratified by the dominant microbial species (Haemophilus, Pseudomonas, and others); B Profiles of ARGs bronchiectasis patients stratified by the dominant microbial species (Haemophilus, Pseudomonas, and others); C Principal coordinate analysis demonstrating the distribution of microbial compositions associated with the Pseudomonas-, Haemophilus-predominant subgroup and the balanced microbial subgroup; D Principal coordinate analysis demonstrating the distribution of ARGs associated with the Pseudomonas-, Haemophilus-predominant subgroup and the balanced microbial subgroup. ARG: antibiotic resistance gene [file 12931_2023_2562_MOESM2_ESM.jpg]

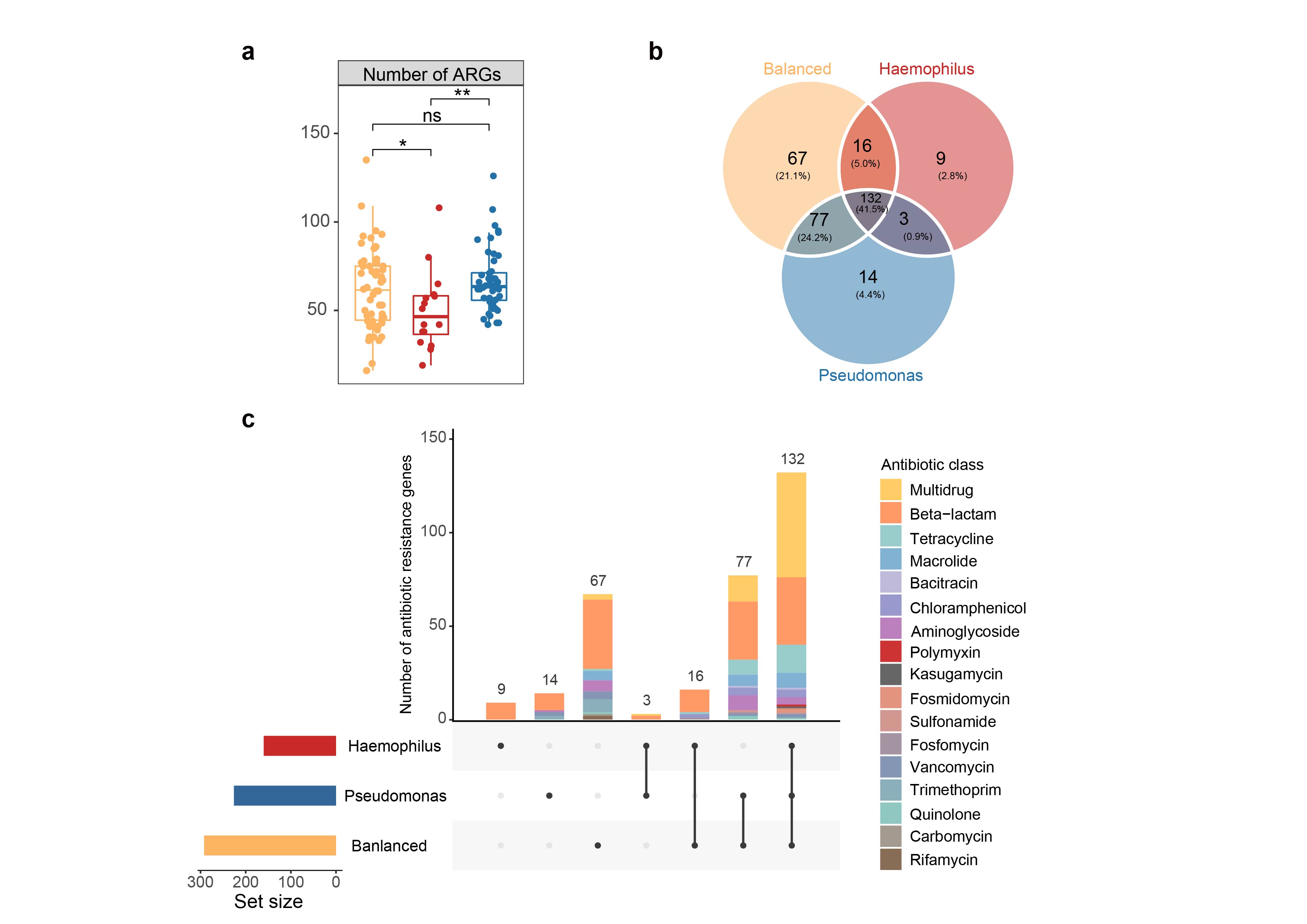

Supplement: Supplementary file 3 — Additional file 3: Figure S3. The spectra of antibiotic resistance genes among bronchiectasis patients with different disease severity and healthy controls. A Box and dot plot comparing the number of ARGs among the Pseudomonas-predominant, Haemophilus-predominant, and a balanced microbiome composition subgroup (other); B The Venn diagram demonstrating the overlap and unique ARGs among bronchiectasis patients with different microbial profiles; C Stack bar chart demonstrating the distribution of ARGs among bronchiectasis patients with different microbial profiles based on the specific categories of antibiotics. ARG: antibiotic resistance gene [file 12931_2023_2562_MOESM3_ESM.jpg]

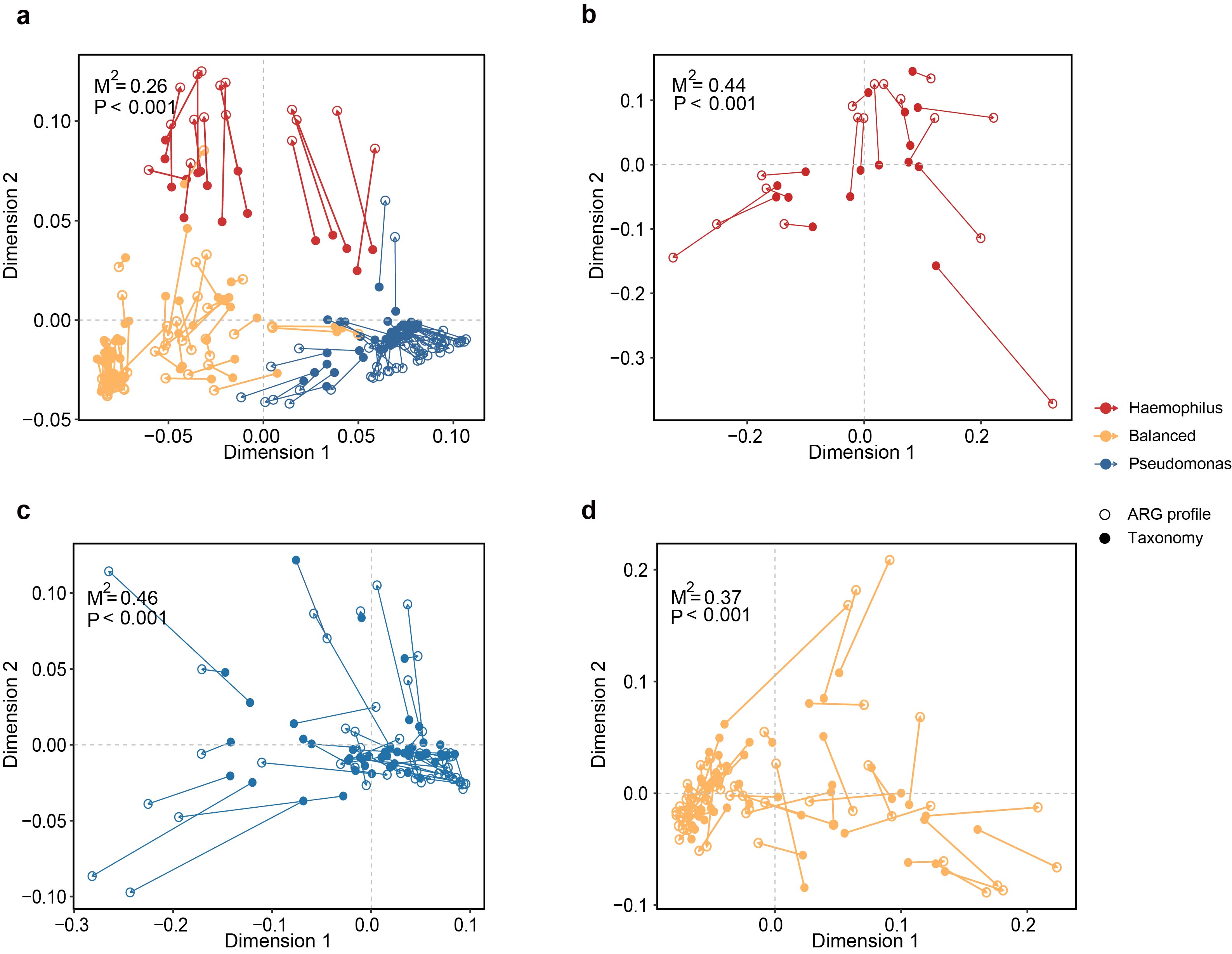

Supplement: Supplementary file 4 — Additional file 4: Figure S4. Correlation between the microbial compositions and the ARG profiles in bronchiectasis patients when clinically stable. A Overall correlation analysis of the three distinct microbial subgroups; B Correlation analysis of the Haemophilus-predominant subgroup; C Correlation analysis of the Pseudomonas-predominant subgroup; D Correlation analysis of the balanced microbial subgroup (other). The Pseudomonas-predominant subgroup was characterized by the Pseudomonas relative abundance of 73.0% ± 20.6%, the Haemophilus–predominant subgroup by the Haemophils relative abundance of 70.8% ± 15.3%, and the balanced microbiota subgroup by no single dominant microbe. [file 12931_2023_2562_MOESM4_ESM.jpg]

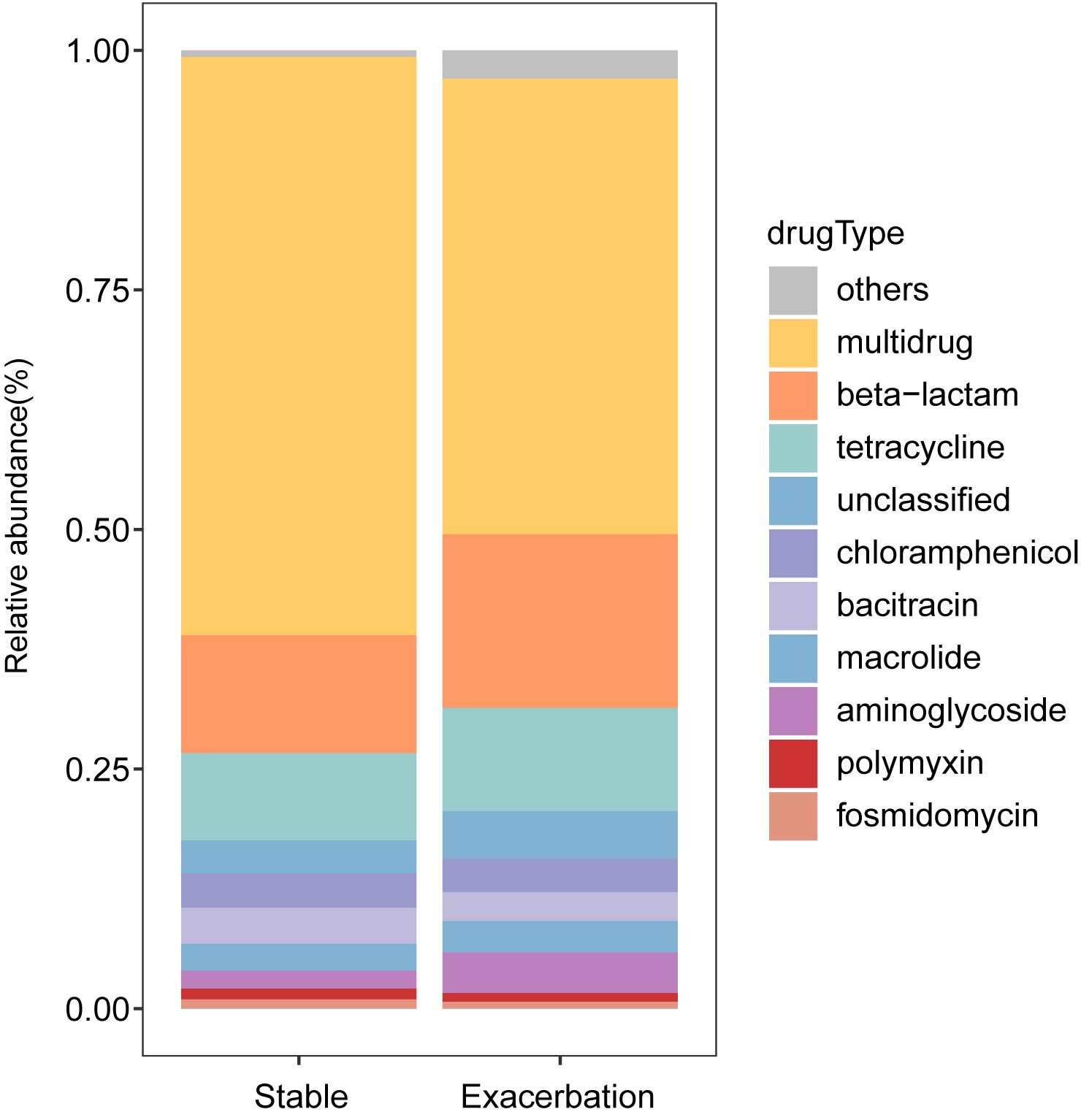

Supplement: Supplementary file 5 — Additional file 5: Figure S5. Representation of ARGs of beta-lactam resistance over multi-drug resistance in bronchiectasis patients when clinically stable and at onset of exacerbations. [file 12931_2023_2562_MOESM5_ESM.jpg]

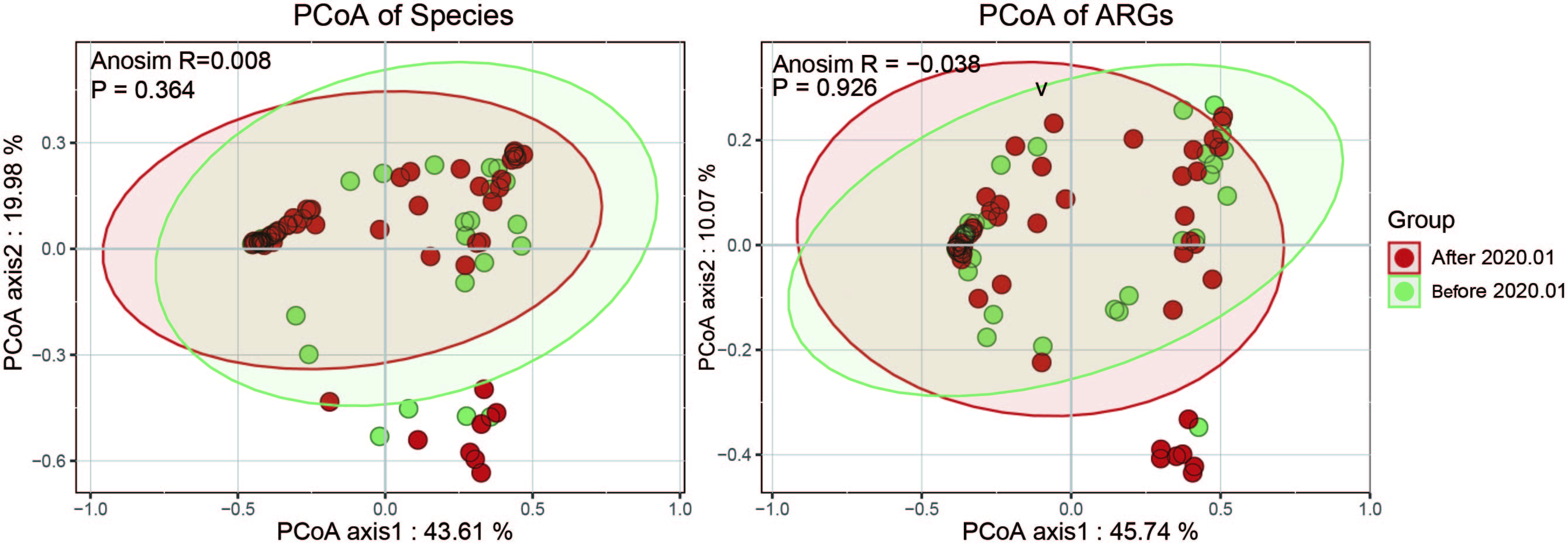

Supplement: Supplementary file 6 — Additional file 6: Figure S6. Comparison of the microbial compositions and ARG profiles between the samples collected prior to and after the COVID-19 outbreak. Shown are the results performed with the principal component analysis and Anosim model. [file 12931_2023_2562_MOESM6_ESM.jpg]

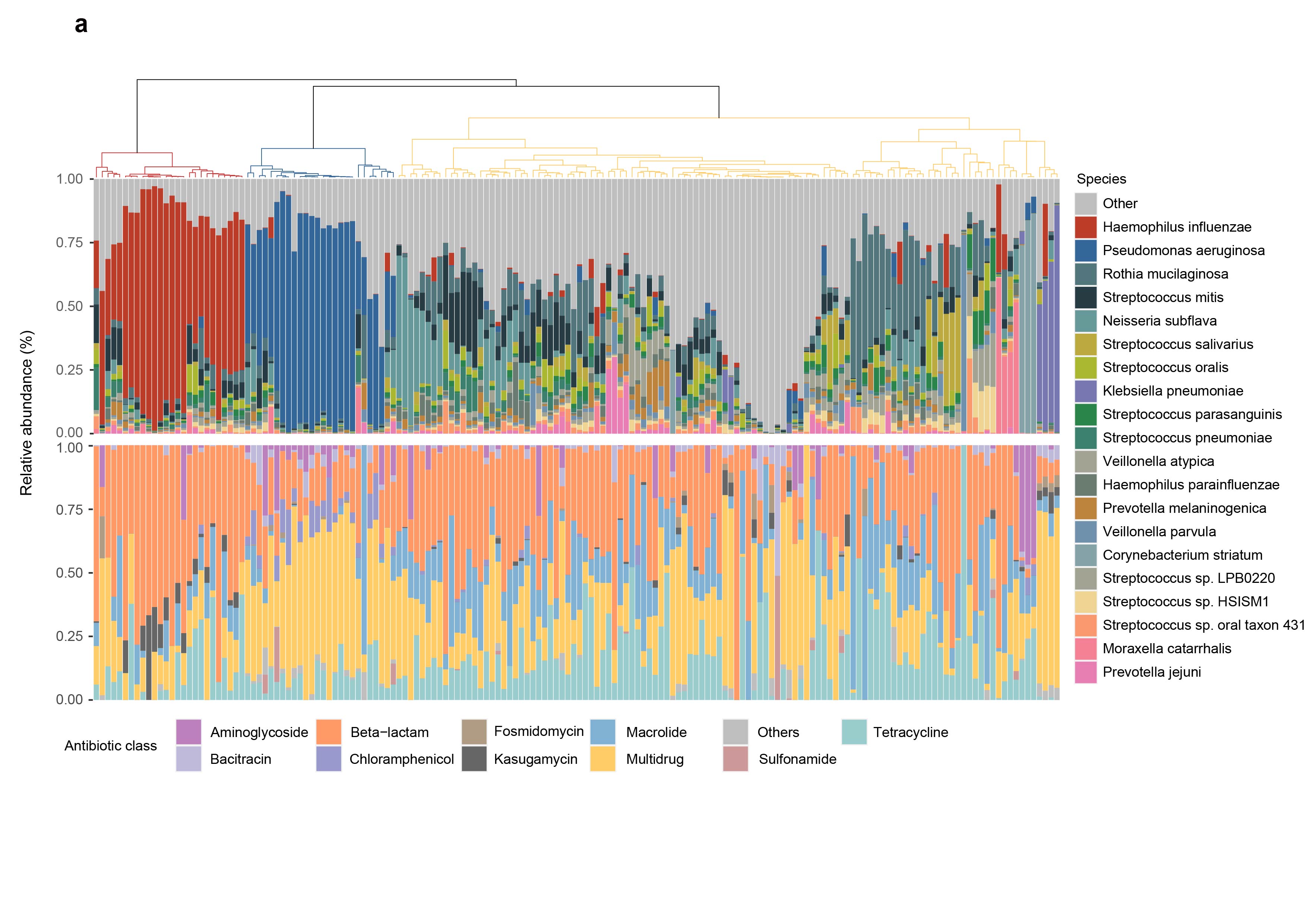

Supplement: Supplementary file 7 — Additional file 7: Figure S7. The sputum resistome in bronchiectasis patients from the Guangzhou cohort and the external validation cohort. Unsupervised clustering revealed three microbiome clusters in the validation dataset. However, there is an over-representation of the balanced microbiota subgroup in the multinational cohort as compared with the Guangzhou cohort. [file 12931_2023_2562_MOESM7_ESM.jpg]
