# Supplementary material for: Differential airway resistome and its correlations with clinical characteristics in Haemophilus- or Pseudomonas-predominant microbial subtypes of bronchiectasis
Source: Respir Res. 2023 Nov 2;24:264. doi: 10.1186/s12931-023-02562-8 (PMC10623730; doi:10.1186/s12931-023-02562-8)
Supplement: Supplementary file 14 — Additional file 14. A word document detailing additional methods, results, and figure and table legends for the Additional files. [file 12931_2023_2562_MOESM14_ESM.docx]

**Online supplement**

**Differential airway resistome and its correlations with clinical characteristics in *Haemophilus-* or *Pseudomonas*-predominant microbial subtypes of bronchiectasis**

**METHODS**

***Definition of acute exacerbation of bronchiectasis***

AEs denoted significant deterioration of three or more symptoms lasting for ≥48hours (cough; sputum volume and/or consistency; sputum purulence; breathlessness and/or exercise tolerance; fatigue and/or malaise; hemoptysis) that required immediate changes in treatment.

***Quality control criteria for sputum plugs***

Fresh sputum was sampled during the out-patient clinics, both when clinically stable and throughout the course of AE. We collected sputum by instructing the patients to forcefully cough up sputum after thoroughly gargling their mouth with distilled water to minimize oral contamination. The sputum plug was dragged repeatedly by using a sterile tweezers on the sterile culture dish to remove saliva. The most purulent component of the sputum plug was selected for microscopic examination without staining. We applied Murray's criteria for the quality control: more than 25 polymorphonuclear and less than 10 epithelial cells under low-power field.

***Sputum cell differentials***

Quality-controlled sputum plugs were split for fresh frozen in -80 degree freezers for subsequent metgenomic sequencing, and the cell differentials with glass smear for haematoxylin-eosin staining. Eosinophilia was defined as having eosinophils that accounted for 3% or more of the sputum inflammatory cell count on low-power fields, while neutrophilia was defined as having neutrophils that accounted for 61% or more of the sputum inflammatory cell count on low-power fields.

**RESULTS**

***Microbial taxa in the DNA extraction blank controls***

A major concern regading the methodology of reporting the microbial compositions for human samples is impact of the artefacts, for instance, the potential contamination due to the reagents or procedures during metagenomic sequencing. To this end, we have sequenced two DNA extraction blank controls along with all of the sputum samples derived from bronchiectasis. According to **Figure S1**, the microbial taxa in the DNA extraction blank controls differed considerably from those in the sputum from patients. This was particularly evident when taking into account the proportion or relative abundance of the dominant bacterial species found in patients with bronchiectasis. Our findings indicated a fairly low likelihood of sample contamination due to the sequencing reagents or the procedures.

**Online Figure legends**

**Figure S1. Comparison of the microbial compositions between two DNA extraction blank controls and the quality-controlled sputum from patients with bronchiectasis**

Shown in the left side of the left and right panels of each figure are the microbial compositions from two DNA extraction blank controls. The samples denote the sputum derived from patients with bronchiectasis. There exist notable differences in the DNA extraction blank controls and the sputum samples, particularly with regard to the dominant microbial taxa identified in patients with bronchiectasis, precluding the major contamination from the sequencing reagents or procedures.

**Figure S2. Comparison of the microbial compositions and ARGs when stratified by the predominant species in patients with bronchiectasis when clinically stable**

1. Microbial profiles of bronchiectasis patients stratified by the dominant microbial species (*Haemophilus*, *Pseudomonas*, and others);
2. Profiles of ARGs bronchiectasis patients stratified by the dominant microbial species (*Haemophilus*, *Pseudomonas*, and others);
3. Principal coordinate analysis demonstrating the distribution of microbial compositions associated with the *Pseudomonas*-, *Haemophilus*-predominant subgroup and the balanced microbial subgroup;
4. Principal coordinate analysis demonstrating the distribution of ARGs associated with the *Pseudomonas*-, *Haemophilus*-predominant subgroup and the balanced microbial subgroup.

ARG: antibiotic resistance gene

**Figure S3. The spectra of antibiotic resistance genes among bronchiectasis patients with different disease severity and healthy controls**

1. Box and dot plot comparing the number of ARGs among the *Pseudomonas*-predominant, *Haemophilus*-predominant, and a balanced microbiome composition subgroup (other);
2. The Venn diagram demonstrating the overlap and unique ARGs among bronchiectasis patients with different microbial profiles;

C) Stack bar chart demonstrating the distribution of ARGs among bronchiectasis patients with different microbial profiles based on the specific categories of antibiotics.

ARG: antibiotic resistance gene

**Figure S4. Correlation between the microbial compositions and the ARG profiles in bronchiectasis patients when clinically stable**

1. Overall correlation analysis of the three distinct microbial subgroups;
2. Correlation analysis of the *Haemophilus*-predominant subgroup;
3. Correlation analysis of the *Pseudomonas*-predominant subgroup;
4. Correlation analysis of the balanced microbial subgroup (other)

*The Pseudomonas*-predominant subgroup was characterized by the *Pseudomonas* relative abundance of 73.0%±20.6%, the *Haemophilus*–predominant subgroup by the *Haemophils* relative abundance of 70.8%±15.3%, and the balanced microbiota subgroup by no single dominant microbe.

**Figure S5. Representation of ARGs of beta-lactam resistance over multi-drug resistance in bronchiectasis patients when clinically stable and at onset of exacerbations**

**Figure S6. Comparison of the microbial compositions and ARG profiles between the samples collected prior to and after the COVID-19 outbreak**

Shown are the results performed with the principal component analysis and Anosim model.

**Figure S7. The sputum resistome in bronchiectasis patients from the Guangzhou cohort and the external validation cohort**

Unsupervised clustering revealed three microbiome clusters in the validation dataset. However, there is an over-representation of the balanced microbiota subgroup in the multinational cohort as compared with the Guangzhou cohort.

**Supplementary Tables**

**Table S1.** The baseline (stable state) clinical characteristics for individuals classified as *Haemophilus*-predominant, *Pseudomonas*-predominant and balanced microbiome subtypes.

**Table S2.** The Adonis associations between clinical parameters and the ARG profiles in our dataset, among all stable samples and within the subgroup of *Haemophilus*-predominant and *Pseudomonas*-predominant respectively.

**Table S3.** The differentially abundant ARGs in comparisons of the *Haemophilus*-predominant and *Pseudomonas*-predominant subgroups versus the balanced microbiota subgroup. Also shown are their statistics in the Mac Aogain et al. dataset.

**Table S4.** The association between clinical parameters and individual ARGs among all stable samples and within the subgroup of *Haemophilus*-predominant and *Pseudomonas*-predominant respectively.

**Table S5.** The Adonis associations between clinical parameters and the ARG profiles in the Mac Aogain et al. dataset, among all stable samples and within the subgroup of *Haemophilus*-predominant and *Pseudomonas*-predominant respectively.

**Table S6.** The associations between the ARGs and clinical parameters that are shared by both our dataset and the Mac Aogain et al. dataset.
